# Influence of the COVID-19 pandemic on births and induced abortions in Southern Sweden: a register-based study

**DOI:** 10.1136/bmjsrh-2023-202162

**Published:** 2024-06-04

**Authors:** Jesse D Thacher, Andreas Vilhelmsson, Annelise J Blomberg, Lars Rylander, Anna Jöud, Lone Schmidt, Charlotte Ørsted Hougaard, Eva Elmerstig, Ditte Vassard, Kristina Mattsson

**Affiliations:** 1 Division of Occupational and Environmental Medicine, Department of Laboratory Medicine, Lund University, Lund, Sweden; 2 Department of Research and Development, Skåne University Hospital, Lund, Sweden; 3 Department of Clinical Sciences Lund, Lund University, Lund, Sweden; 4 Department of Public Health, University of Copenhagen, Copenhagen, Denmark; 5 Department of Social Work, Centre for Sexology and Sexuality Studies, Malmö University, Malmö, Sweden; 6 Department of Obstetrics and Gynecology, Skåne University Hospital, Lund, Sweden

**Keywords:** COVID-19, abortion, induced, epidemiology, Reproductive Behavior

## Abstract

**ABSTRACT:**

**Background:**

Pandemics are linked with declining birth rates, but little is known about how the COVID-19 pandemic has influenced childbearing decisions. We aimed to investigate the associations between the COVID-19 pandemic and reproductive decisions, specifically to identify potential changes in the frequency of deliveries and induced abortions in Skåne, Sweden.

**Methods:**

Using the Skåne Healthcare Register, we identified women aged 15–45 years who had at least one pregnancy-related care visit registered between 1 January 2013 and 11 November 11 2021. Deliveries and induced abortions were identified, and changes in weekly delivery and abortion counts were assessed using an interrupted time series design. Relative risks (RRs) and 95% confidence intervals (CIs) were estimated from a Poisson regression model.

**Results:**

During the study period we identified 129 131 deliveries and 38 591 abortions. Compared with the counterfactual (exposed interval assuming COVID-19 had not occurred), pandemic exposure was associated with fewer deliveries (RR 0.93; 95% CI 0.89 to 0.98). For abortions, pandemic exposure appeared to be associated with fewer abortions (RR 0.95; 95% CI 0.90 to 1.00); however, age-related differences were found. Among women aged 25 years and over, pandemic exposure was more strongly associated with fewer abortions. Contrastingly, among women aged under 25 years, abortions appeared to increase.

**Conclusions:**

The COVID-19 pandemic seemed to have contributed to a decline in births in Southern Sweden. During the same period, abortions declined in women in the older age range, but contrastingly increased among younger women.

**WHAT IS ALREADY KNOWN ON THIS TOPIC**

- Earlier pandemics have generally been associated with reduced birth rates following their peaks, but little is known about how pandemics influence induced abortion rates.

**WHAT THIS STUDY ADDS**

- The COVID-19 pandemic seemed to have contributed to a decline in births in Southern Sweden. Additionally, there appeared to be a decline in abortions among women aged 25 years and over, but contrastingly an increase in abortions in younger women.

**HOW THIS STUDY MIGHT AFFECT RESEARCH, PRACTICE OR POLICY**

- The present findings suggest that the pandemic influenced reproductive decisions, but not necessarily in the same manner for women of different ages. This highlights the need for further studies to better understand reproductive choices in times of crisis.

## Introduction

 Societal crises have historically led to a reduction in childbearing.^1 2^ Spikes in mortality due to wars, famines, natural disasters and pandemics were followed by fewer births in the short term and recovery in subsequent years.^3 4^ Previous pandemics, such as the 1918 H1N1, the 2013 Ebola and the 2016 Zika virus outbreaks were associated with declining birth rates about 9 months following epidemic peaks.^2 5^ Reasons for these declines included high parental mortality (H1N1 and Ebola) or high fetal morbidity and mortality (Zika).^6^

However, increased mortality in cases of direct exposure is not the only demographic consequence of a pandemic, as birth rates may also be affected through indirect effects related to social fears, distancing measures, relationship challenges, as well as social and financial circumstances causing couples to postpone pregnancy in times of crisis.^5 7 8^

As with previous pandemics, the COVID-19 outbreak in 2020 was associated with a decline in births 9 months after its first wave in many countries. For example, of 24 investigated European countries, 78.5% experienced a decline in live births, ranging between −0.5% and −11.4%, with the most pronounced decline in countries with the highest degree of excess mortality.^2^ However, on a national level, Sweden experienced no changes to overall birth rates.^2^ The COVID-19 pandemic led to a multitier crisis disrupting not only global health, but also social welfare and the economy, leading to stressful and insecure conditions across many nations.^9^ Access to and delivery of healthcare services were also highly affected in many countries.^10^

Overall, little is known in Sweden regarding postponed pregnancies due to the COVID-19 pandemic, with no studies investigating long-term trends utilising individual level time series data. Nonetheless, some smaller studies have been published. For instance, a Swedish study investigated if the quarterly number of induced abortions and ongoing pregnancies changed during the first pandemic wave of COVID-19 in 2020, by combining aggregated register data on abortions with interview data from women seeking abortion care.^11^ The study observed no changes in the number of abortions and ongoing pregnancies compared with the previous 2 years. Additionally, a questionnaire-based study of 600 women seeking a first-trimester abortion in different parts of Sweden reported that approximately 13% stated that the pandemic had affected their decision, with 11% reporting it affected them somewhat and 2% reporting it affected them a lot. Since the pandemic began, 6% stated that COVID-19 was the main reason for their abortion.^12^ Elsewhere in Europe, a British survey of 5733 participants observed fewer reported abortions in the first year of the pandemic compared with data collected a decade earlier (2010–2012).^13^

Understanding the changes in birth and abortion rates is important, as changes can have significant consequences on demographic pyramids. This is even more critical in countries with an already low number of children per couple such as Sweden. Furthermore, such knowledge helps inform resource allocation and adaptations in future crisis planning. To date, no register-based Swedish study with individual-level data has assessed the number of births and abortions accounting for trends before, during and after the onset of the COVID-19 pandemic using 9 years of time series data. We hypothesised that similar to comparable countries and previous crises, both deliveries and abortions would have decreased during the COVID-19 pandemic. Therefore, we aimed to investigate associations between the COVID-19 pandemic and reproductive decisions, specifically to identify potential changes in the frequency of deliveries and abortions in Skåne, Sweden, comparing the pre-pandemic and pandemic periods.

## Methods

The study base was a regional cohort of all women aged 15–45 years who had at least one pregnancy-associated care visit registered between 1 January 2013 and 11 November 2021 in the regional database of the Southern county of Sweden (Skåne): the Skåne Healthcare Register (SHR).^14^ Skåne had a population of 1 377 827 at the beginning of 2020, representing 13% of the country’s population.

The SHR is an administrative healthcare register which compiles information transferred from administrative application sources on all healthcare consultations in Skåne from 1998 onwards. Each interaction, both somatic and psychiatric, from all types of healthcare professionals (physician, nurse, physiotherapists, etc.) generate data entries by the healthcare provider that are then automatically transferred to the SHR. These entries constitute the basis for economic reimbursement to the healthcare provider, and coverage in the SHR has been shown to be close to 100% for inpatient care.^14^

The available data include age, sex, hospital admission and discharge dates, healthcare provider, clinic, deaths and diagnostic codes according to the International Classification of Diseases, Tenth Revision (ICD-10).

### Outcome definition

Up to 15 ICD-10 diagnostic codes for each consultation can be registered in the SHR, and each interaction is registered with one primary diagnosis and one or more secondary diagnoses. In tertiary inpatient care, the first registered diagnosis must describe the principal reason for the consultation. Pregnancy-associated care visits were identified through ICD-10 codes, specifically Chapter O on pregnancy, childbirth and the puerperium. Deliveries and abortions were defined using the primary diagnosis. Deliveries were defined as O80-O84, and abortions were defined as O04-O06. In Sweden, women have free right of an abortion up to gestational week 18 (17+6), and after approval from the National Board of Health and Welfare’s judicial council up until week 21+6. Abortion care is provided through the public healthcare system; the gestational week and, to some extent, the woman’s preference, determine which treatment method is used. If several pregnancy-associated interactions were detected, a “washout” period of 90 days was required before an additional abortion could be included, and 210 days (30 weeks) before an additional delivery could be included. All relevant ICD-10 codes relevant to pregnancy were identified to compile a scheme of relevant codes (online supplemental figure S1).

### Statistical methods

We implemented an interrupted time series study design and included all pregnant women in Southern Sweden whose information appeared in the Skåne Healthcare Register (SHR) from 1 January 2013 to 11 November 2021.^15 16^

The interval before 11 March 2020 (start of first COVID-19 wave in Sweden) was classified as unexposed to the pandemic and the interval beginning on that date was classified as exposed. Our primary analyses quantified the association of the COVID-19 pandemic with the weekly delivery and abortion counts. Relative risks (RRs) and 95% confidence intervals (CIs) were estimated using a Poisson regression model.

Specifically, to compare the pre- and post-COVID-19 periods accounting for underlying trends, we modelled the weekly delivery and abortion counts by fitting a Poisson regression model with the following covariates: time (with the week as the underlying time unit) and the COVID-19 exposure interaction with time (where exposure was classified as unexposed or exposed to the COVID-19 pandemic). We used Fourier terms in the Poisson model as covariates to model the seasonal component.^15^ Further model details can be found in the online supplemental material (Model Details).

In subgroup analyses, we stratified our sample based on age in the following three groups, ages 15–24, 25–34 and ≥35 years. Additionally, as a sensitivity analysis, we examined the change in weekly abortion rates (ie, the number of abortions divided by the sum of births and abortions in each week) comparing the pre- and post-COVID-19 periods. To examine the effect of truncation bias, we ended follow-up on 30 September 2021 and repeated the analyses. Lastly, as an additional sensitivity analysis, we tested using a later exposed and unexposed interval, using an interruption date of 1 September 2020 for deliveries and 1 April 2020 for abortions.

Statistical analyses were performed in SAS 9.4 (SAS Institute, Cary, NC, USA) and R version 4.2.2 including R package ‘its2es’.

The study was approved by the Swedish Ethical Review Authority (Reg. No. 2021–01798).

### Patient and public involvement

Patients and/or the public were not directly involved in the design, conduct, reporting, or dissemination plans of this research.

## Results

From the period 1 January 2013 to 11 November 2021, we identified 129 131 deliveries and 38 591 abortions among women aged 15–45 years. Online supplemental figure S2 illustrates the overall birth and abortion counts in Skåne over the study period.

**Figure 1:**
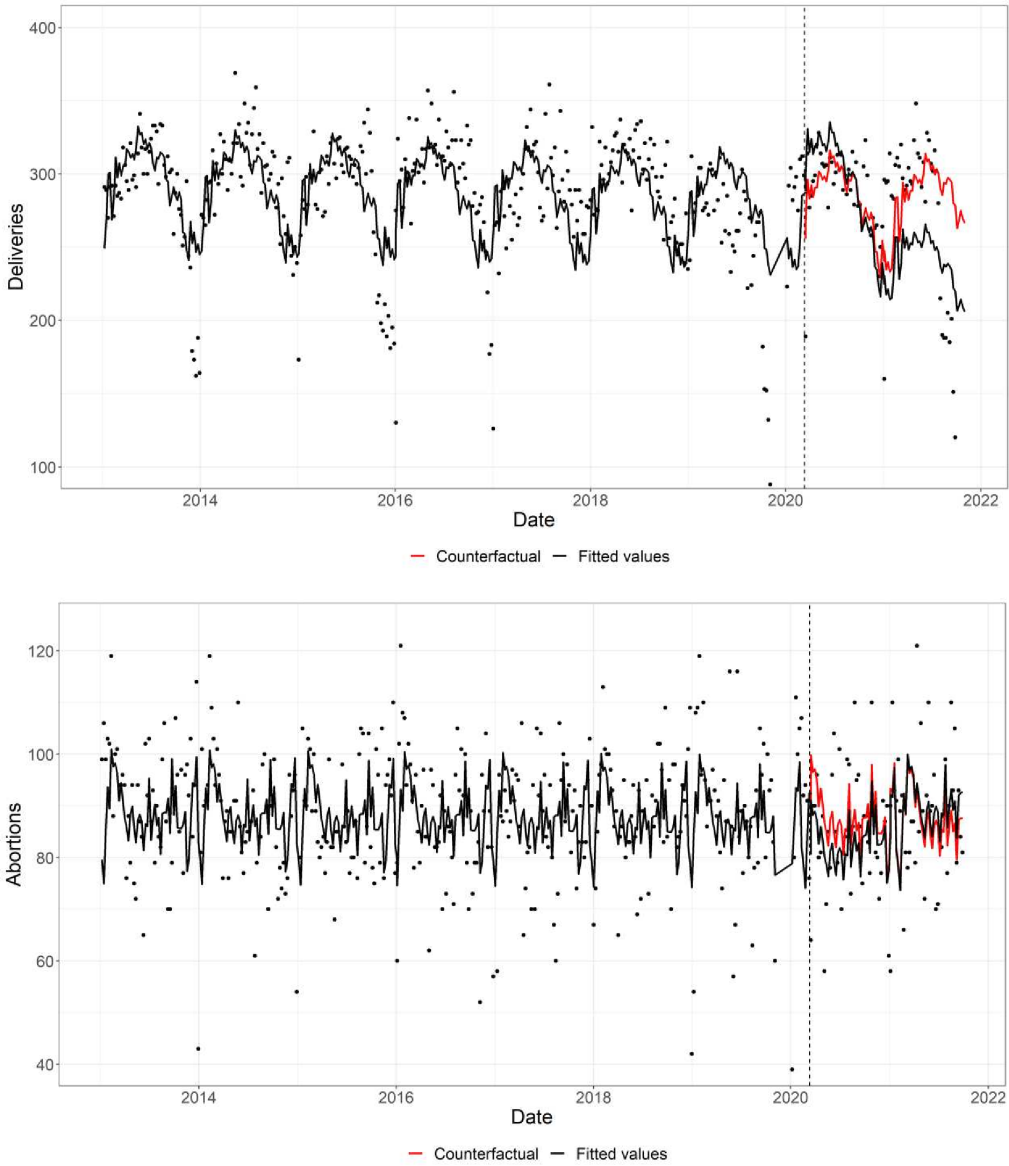
The weekly deliveries (top) and induced abortions (bottom) modelled using a Poisson regression with seasonal adjustments. The counterfactual refers to the predicted values had no COVID-19 pandemic occurred, and the fitted values are estimated based on the regression model. Grey dotted line represents 11 March 2020 (interruption).

**Figure 2:**
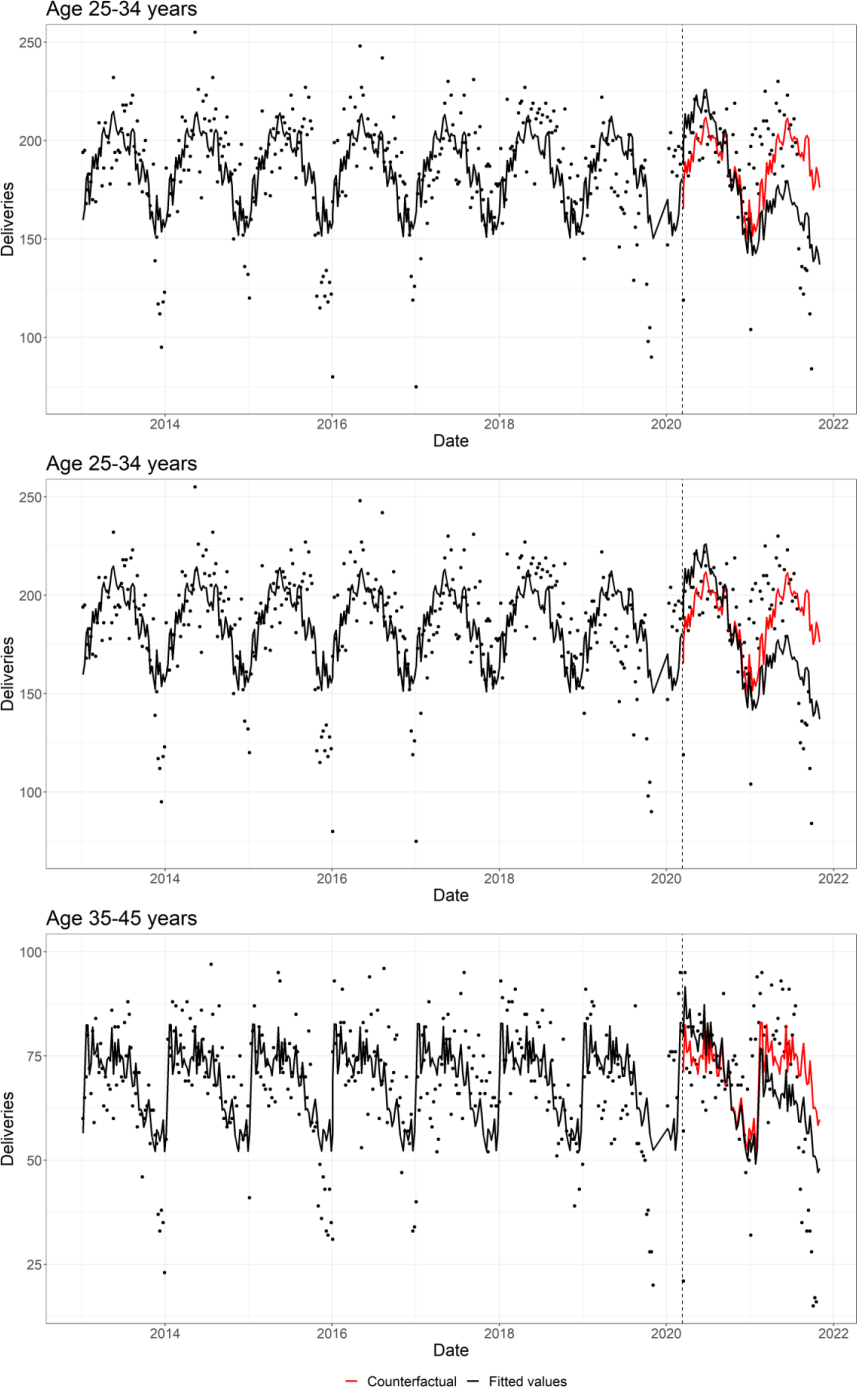
Scatter plot of the weekly deliveries by age group, together with the regression fitted values (in black) and the counterfactual (in red). Grey dotted line represents 11 March 2020 (interruption).

In principal analyses, compared with the counterfactual (the exposed interval assuming COVID-19 had not occurred), pandemic exposure was associated with fewer deliveries (RR 0.93; 95% CI 0.89 to 0.98) (table 1, figure 1). That is, there was a reduction in births of about 7%. The reduction in deliveries tended to be more pronounced among women under the age of 35 years, particularly those under 25 years (RR 0.81; 95% CI 0.74 to 0.88) (table 1, figure 2).

**Table 1:** Relative risk of the weekly deliveries and abortions, comparing the fitted values with the model-based counterfactual values

Type of analysis	DeliveriesRR (95% CI)	P value	AbortionsRR (95% CI)	P value
Primary analysis*	0.93 (0.89 to 0.98)	0.01	0.95 (0.90 to 1.00)	0.04
Slope change	0.89 (0.85 to 0.92)	<0.001	0.97 (0.93 to 1.01)	0.14
Level change	0.95 (0.90 to 1.00)	0.06	0.95 (0.90 to 1.00)	0.04
Sensitivity analysis of demographic factors*				
Women aged 15–24 years	0.81 (0.74 to 0.88)	<0.001	1.06 (0.98 to 1.15)	0.17
Women aged 25–34 years	0.94 (0.89 to 0.99)	0.03	0.89 (0.84 to 0.95)	<0.001
Women aged 35–45 years	0.95 (0.89 to 1.01)	0.11	0.91 (0.84 to 0.99)	0.03

For each exposure, the reference group was the model-based counterfactual values, assuming that the COVID-19 pandemic had not occurred.

* Model for slope and level change adjusted for seasonal effects.

CIconfidence intervalRRrelative risk

**Figure 3:**
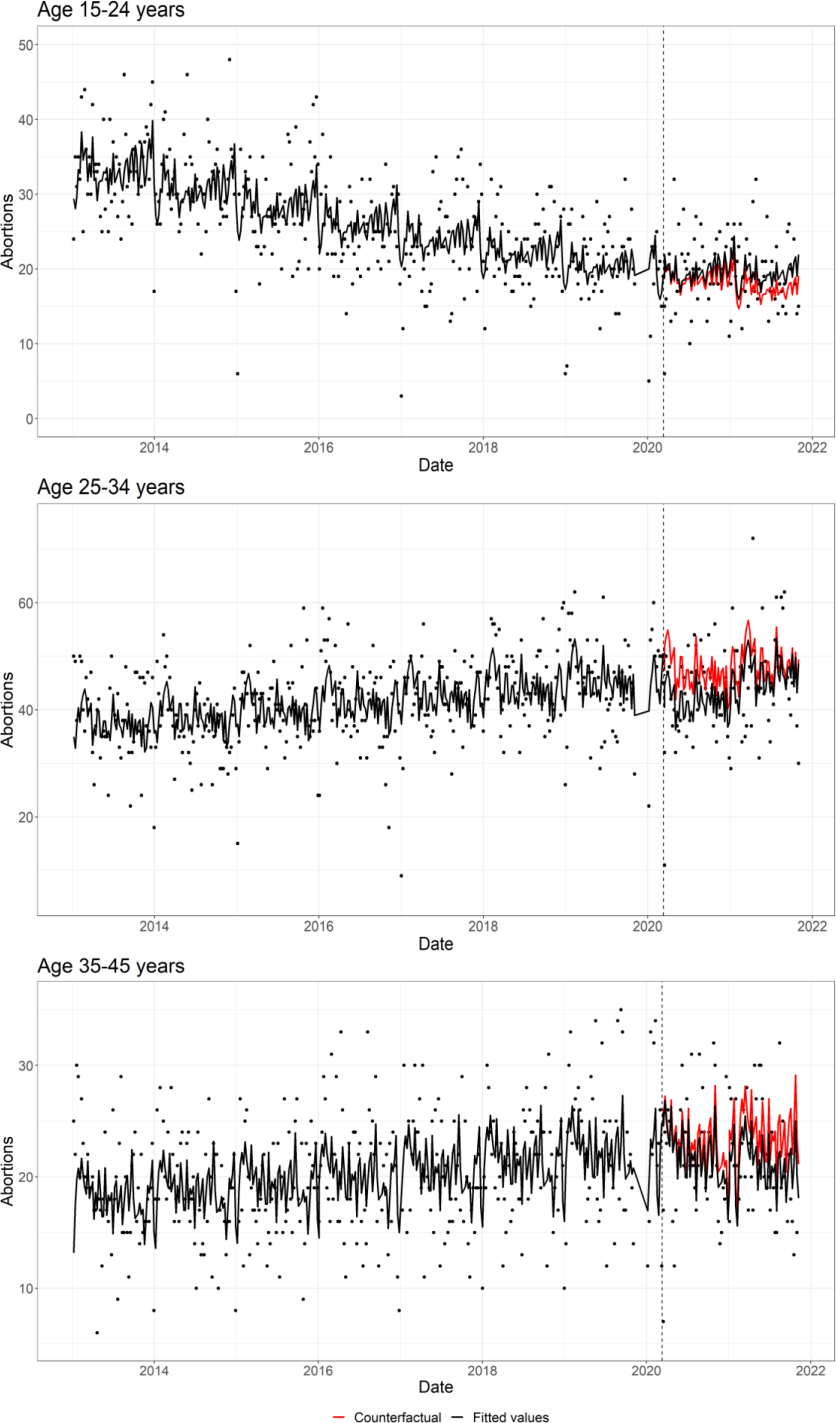
Scatter plot of the weekly induced abortions by age group, together with the regression fitted values (in black) and the counterfactual (in red). Grey dotted line represents 11 March 2020 (interruption).

For abortions, among all women aged 15–45 years, we observed some indication that pandemic exposure was associated with fewer abortions (RR 0.95; 95% CI 0.90 to 1.00) (table 1, figure 1). In the older age groups, pandemic exposure was more strongly associated with fewer abortions (RRs of 0.89 (95% CI 0.84 to 0.95) and 0.91 (95% CI 0.84 to 0.99) for women aged 25–34 and 35–45 years, respectively) (table 1, figure 3). Contrastingly, there was some suggestion that abortion counts among women aged 15–24 years were more frequent compared with the pre-pandemic period (RR 1.06; 95% CI 0.98 to 1.15). Moreover, when we examined abortion rates (ie, number of abortions divided by the sum of abortions plus deliveries), we found that in this younger age group, pandemic exposure was associated with a higher abortion rate (RR 1.12; 95% CI 1.05 to 1.19) (online supplemental table S1).

In sensitivity analyses, ending follow-up early drove the overall risk estimates for deliveries and abortions towards unity. However, in subgroup analyses, only small changes were noted for births and abortions (online supplemental table S2). Changing the date for the exposed and unexposed interval generally led to small changes in overall risk estimates; for births the RR was 0.92 (95% CI 0.87 to 0.97) and for abortions the RR was 1.01 (95% CI 0.96 to 1.06).

## Discussion

Overall, our findings indicate that the frequency of deliveries seemed to decrease compared with pre-pandemic years. Similarly, a tendency of fewer abortions was noted, particularly among women aged 25 years and over. Contrastingly, younger women (aged 15–24 years) appeared to have more abortions.

Only a handful of studies have investigated the impact of COVID-19 on deliveries or abortions. Our findings are congruent with previous studies on deliveries, showing a decline in birth rates following the COVID-19 pandemic.^2 17 18^ In a study of 14 European countries, a 14% decline in births 9 months following the first COVID-19 wave was reported.^2^ Similarly, a US-based study demonstrated that the COVID-19 pandemic was associated with a national decline in fertility rates.^18^ Lastly, a questionnaire-based UK study showed that the COVID-19 pandemic influenced pregnancy-planning behaviours, with many women reporting postponement of pregnancy.^17^

Even less is known about how the COVID-19 pandemic has influenced abortions. Our findings conflict with an earlier Swedish study which reported that the national abortion rates remained unchanged during the first wave of the COVID-19 pandemic compared with the two previous years.^11^ However, this study was based on quarterly abortion rates and only comprised data voluntarily reported from clinics performing abortions from 2018 to 2020. Moreover, a publication from the Swedish Board of Health and Welfare, including data from later years, reported that the number of abortions in Sweden in 2021 was the lowest since 2002.^19^ Similarly, a British study observed fewer reported abortions in the first year of the pandemic compared with data collected a decade earlier.^13^ We also observed some indication of fewer abortions, particularly among women over 25 years of age. Contrastingly, we found that abortions appeared to increase somewhat among young women, which warrants further investigation.

The reason behind the general trend of fewer abortions could be due to several factors. Concerns such as stress, social isolation and unemployment could be driving forces in pregnancy intention, particularly for a younger demographic. Furthermore, anxiety, restrictions on socialising, as well as relationship challenges related to changing life circumstances, and less sexual contact during the pandemic may have led to lower pregnancy rates, hence reducing the risk of unplanned pregnancies and subsequent abortions.^7 8^ Economic worries, coupled with the absence of sufficient knowledge regarding potential harmful impact on fetal development and the health risks for pregnant women due to COVID-19 infection, could also have influenced couples to delay conception or terminate a pregnancy. The understanding that heightened parental stress, in some studies, has been linked to reduced fertility might contribute to the postponement of conception during times of crisis.^20^ Nevertheless, compared with 23 Organisation for Economic Co-operation and Development (OECD) countries, less pronounced effects of unemployment on fertility were reported in countries with a larger social safety nets such as Sweden and Norway.^21^

Another potential driver behind the reduced abortion numbers regards access to abortion healthcare. In a survey conducted early in the pandemic in 29 countries across the world, access to abortion was limited in approximately half of all countries.^22^ In the aforementioned Swedish study, Rydelius and colleagues found that the proportion of surgical abortions decreased, whereas medical home abortions increased during the study period.^11^ This could reflect surgical resources being sparse during the pandemic; however, secular trends have demonstrated an already ongoing shift from surgical abortions towards home abortions, also for other reasons.^23^ The finding that younger women seemed to have more abortions also speaks against reduced access to care as an important contributing factor. Overall, the measures of abortions in themselves may not give precise clarity on what the health needs of the population may be, potentially capturing competing effects which differ across age groups as well as markers of inequality.^24^

The pandemic’s prolonged stressors on societies with variants of the virus emerging leading to new waves of outbreaks, restrictions and uncertainty of the future,^25^ may have had an impact on reproductive decisions in a longer perspective compared with previous pandemics. As such, future studies might further assess the pandemic, as well as different public health policies’ influence on pregnancy decisions. Furthermore, qualitative studies could elucidate the determinants of reproductive choices during the COVID-19 pandemic.

One of the major strengths of our study is the SHR register, which includes detailed information on, in principle, all births and induced abortions in Skåne, Sweden. The SHR registry is unique as it contains information on abortions in Skåne, both those overseen by midwives as well as abortions in which a physician is involved. Another key strength is the ability to include all pregnancies and abortions in Skåne from 2013 to 2019 (before COVID-19) to establish and control for any potential contemporaneous trends prior to onset of the pandemic. Lastly, our study benefitted from being an entirely register-based study in a country with free healthcare, limiting the potential for bias related to participation or access to healthcare.

The SHR is a unique database, particularly for abortion data, but as an administrative registry it lacks many individual-level confounders. This limitation prevented additional multivariable models and the identification of other potential factors contributing to changes in births or abortions. Truncation bias could affect our study. However, data are available until November 2022, which allowed us to estimate all deliveries and induced abortions more than a year after the onset of the pandemic. Moreover, ending follow-up early demonstrated similar trends, particularly in subgroup analyses. Lastly, the selection of exposed and unexposed interval was somewhat arbitrary; nevertheless, the date coincides well with the first wave of COVID in Sweden and changing the date had minimal impact on the results.

In conclusion, the COVID-19 pandemic seemed to have contributed to a decline in births in Southern Sweden. Additionally, there appeared to be a decline in abortions among women over the age of 24 years and an increase in abortions in women under the age of 25 years. However, further research with additional post-pandemic follow-up is necessary before firm conclusions can be drawn.

## supplementary material

10.1136/bmjsrh-2023-202162 online supplemental material 1

## Data Availability

Data may be obtained from a third party and are not publicly available.
