# Supplementary material for: Influence of the COVID-19 pandemic on births and induced abortions in Southern Sweden: a register-based study
Source: BMJ Sex Reprod Health. 2024 Jun 4;51(1):e202162. doi: 10.1136/bmjsrh-2023-202162 (PMC11874269; doi:10.1136/bmjsrh-2023-202162)

SUPPLEMENTAL MATERIAL

Influence of the COVID-19 pandemic on births and abortions in Southern Sweden: a register-based study

---

Model Details: We modelled the weekly delivery and abortion counts by fitting a Poisson regression model with the following covariates: time (with the week as the underlying time unit), and the COVID-19 exposure interaction with time (where exposure was classified as unexposed or exposed to the COVID-19 pandemic). We used Fourier terms in the Poisson model as covariates to model the seasonal component. The Poisson regression model can be represented using the following notation: denote by t the time (a sequence of weeks from 1 to 458, with $t* = 370$ for 11, March 2020), by Yt the weekly delivery/abortion count, by $Xt = 1(t \geq 370)$ the COVID-19 indicator, and by St the seasonal component (Fourier terms). Therefore, our regression model is as follows:

$$\log(E(Yt \mid Xt, St)) = \beta 0 + \beta 1 \cdot t + \beta 2 \cdot Xt \cdot (t - t*) + \gamma TSt$$

where $\beta 0$ is the intercept, $\beta 1$ is the time trend coefficient, $\beta 2$ is the slope change following the intervention. We then followed recommendations in the literature to estimate the relative risk of the COVID-19 period on deliveries and abortions.

Table S1. Relative risk (RR) of the weekly abortion rates[a], comparing the fitted values with the model-based counterfactual values.

Type of analysis	Overall proportion	Abortion rates RR (95% CI)	P-value
**Primary analysis[a]**	24%	0.99 (0.94-1.04)	0.66
**Sensitivity analysis of demographic factors[b]**			
**Females age 15-24 years**	45%	1.12 (1.05-1.19)	<0.001
**Females age 25-34 years**	19%	0.93 (0.87-0.99)	0.02
**Females age 35-45 years**	23%	0.96 (0.89-1.04)	0.30

[a] The number of abortions divided by the sum of births and abortions in a given week.
[b] Model for slope and level change adjusted for seasonal effects.
For each exposure, the reference group was the model-based counterfactual values, assuming that COVID-19 had not occurred.

Table S2. Relative risk (RR) of the weekly deliveries and abortions, comparing the fitted values with the model-based counterfactual values ending follow-up 30th September 2021.

Type of analysis	Deliveries RR (95% CI)	P-value	Abortions RR (95% CI)	P-value
**Primary analysis**[a]	0.98 (0.94-1.02)	0.48	0.98 (0.93-1.03)	0.48
**Slope change**	0.96 (0.93-0.99)	0.04	1.02 (0.98-1.06)	0.29
**Level change**	0.99 (0.95-1.03)	0.62	0.98 (0.93-1.03)	0.39
**Sensitivity analysis of demographic factors**[a]				
**Females age 15-24 years**	0.85 (0.79-0.92)	<0.001	1.08 (1.00-1.17)	0.06
**Females age 25-34 years**	0.99 (0.95-1.04)	0.80	0.92 (0.87-0.98)	0.01
**Females age 35-45 years**	1.00 (0.94-1.06)	0.90	0.95 (0.88-1.03)	0.19

[a] Model for slope and level change adjusted for seasonal effects.
For each exposure, the reference group was the model-based counterfactual values, assuming that COVID-19 had not occurred.

Figure S1. Procedure of identification of deliveries and abortions in Sweden.

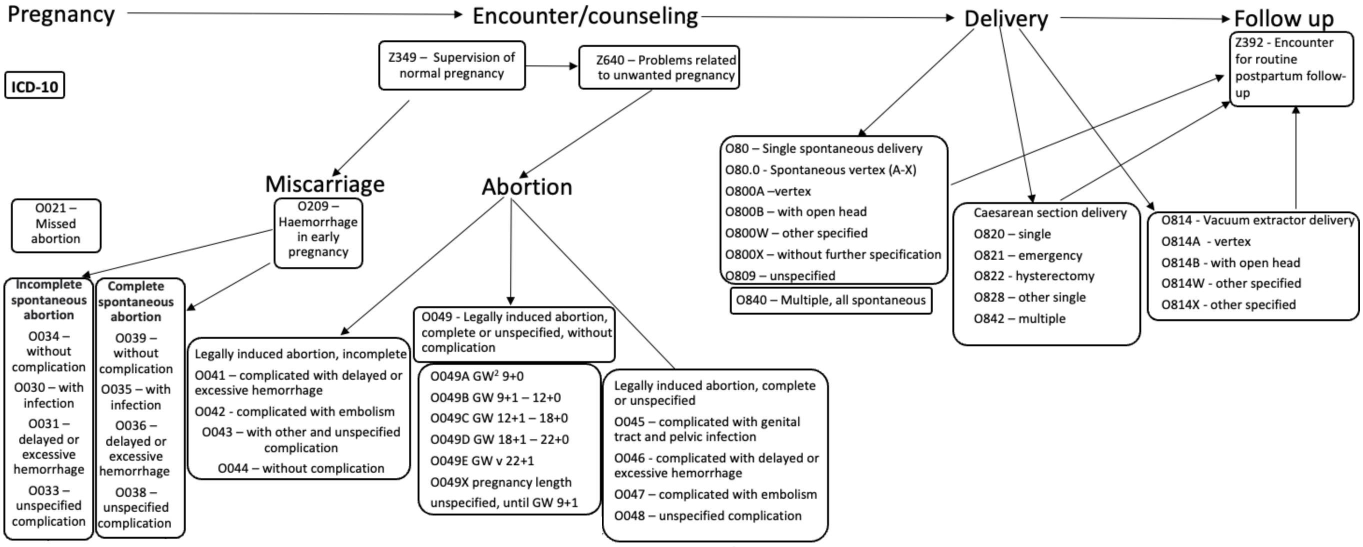

Figure S2. Number of weekly deliveries and abortions in Skåne from January 1, 2013 to November 11, 2021 (end of follow-up). Black dashed line represents March 11th, 2020.

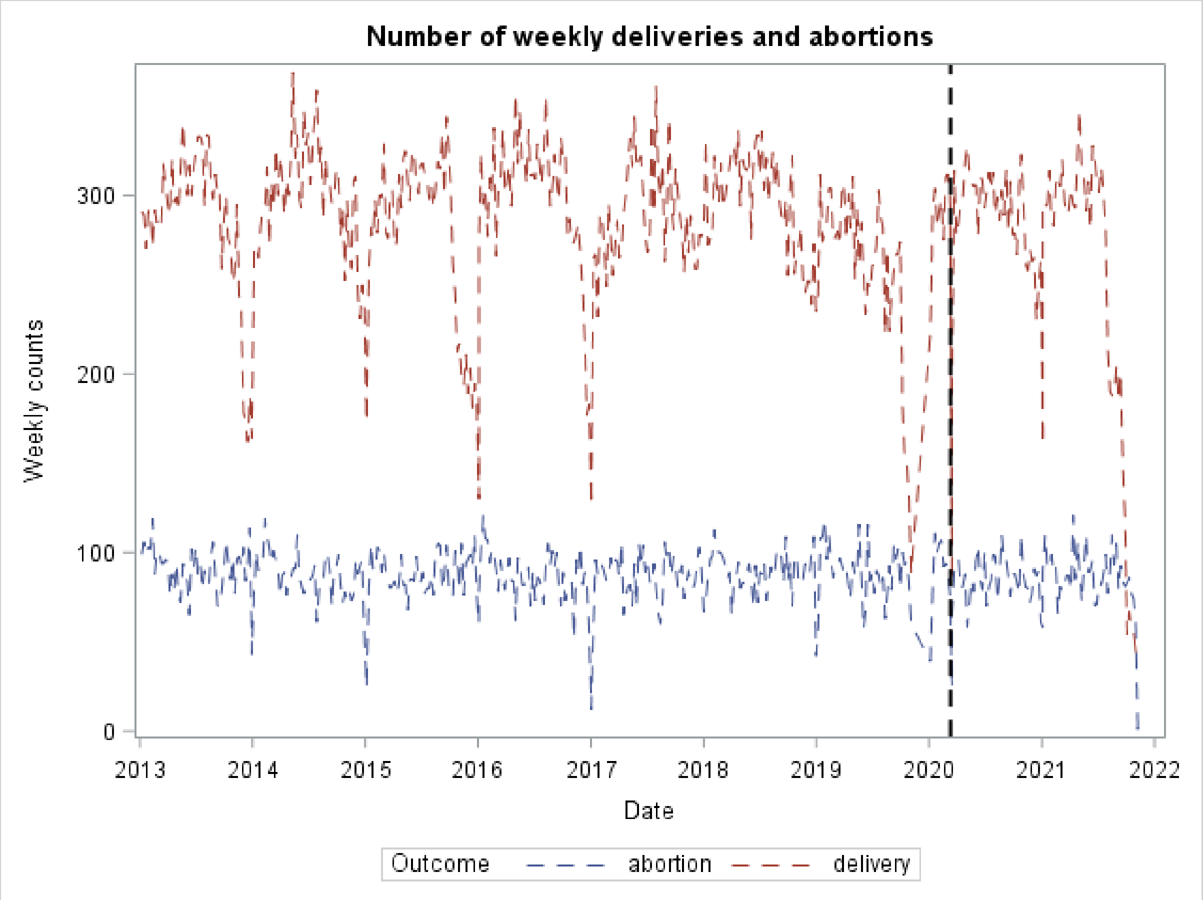

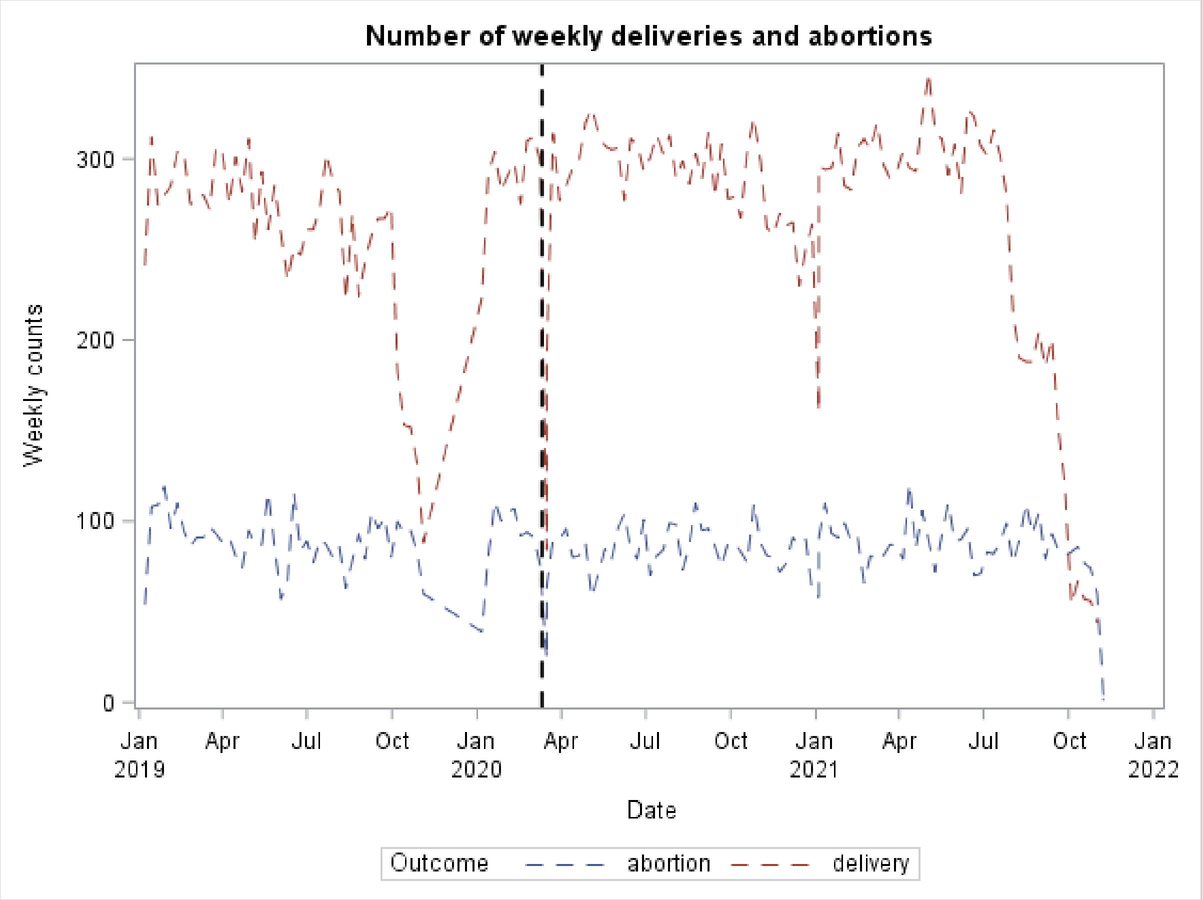

Supplement: online supplemental material 1 [file bmjsrh-51-1-s001.pdf]
